# Effects of Supplementation with Folic Acid and Its Combinations with Other Nutrients on Cognitive Impairment and Alzheimer’s Disease: A Narrative Review

**DOI:** 10.3390/nu13092966

**Published:** 2021-08-26

**Authors:** Ana M. Puga, Mar Ruperto, Mª de Lourdes Samaniego-Vaesken, Ana Montero-Bravo, Teresa Partearroyo, Gregorio Varela-Moreiras

**Affiliations:** 1 Departamento de Ciencias Farmacéuticas y de la Salud, Facultad de Farmacia, Universidad San Pablo-CEU, CEU Universities, Urbanización Montepríncipe, Alcorcón, 28925 Madrid, Spain; anamaria.pugagimenezazca@ceu.es (A.M.P.); mmar.rupertolopez@ceu.es (M.R.); l.samaniego@ceu.es (M.d.L.S.-V.); amontero.fcex@ceu.es (A.M.-B.); t.partearroyo@ceu.es (T.P.); 2 Grupo USP-CEU de Excelencia “Nutrición para la vida (Nutrition for Life)”, ref: E02/0720, Alcorcón, 28925 Madrid, Spain

**Keywords:** Alzheimer’s disease, vascular dementia, mild cognitive impairment, vitamin supplementation, folic acid, cognitive function

## Abstract

Cognitive impairment and Alzheimer’s Disease, among other cognitive dysfunctions, has been recognized as a major public health problem. Folic acid is a well-known essential nutrient whose deficiency has been linked to neurocognitive dysfunctions, owing to hyperhomocysteinemia, an independent risk factor for cardio- and cerebrovascular diseases, including cognitive impairment, Alzheimer’s Disease, and vascular dementia. However, to date, there is certain controversy about the efficacy of vitamin supplementation in patients with these pathologies. Therefore, we have reviewed the available dietary intervention studies based on folic acid, either alone or in combination with different vitamins or nutrients into the progression of Alzheimer’s Disease and Cognitive impairment, highlighting the cognition and biochemical markers employed for the evaluation of the disease progression. Undeniably, the compiled information supports the potential benefits of vitamin supplementation in these pathologies, especially relevant to the aging process and quality of life, although more research is urgently needed to confirm these positive findings.

## 1. Introduction

Undoubtedly, one of the main challenges to be faced in the upcoming years is population ageing. This implies that the increase in the prevalence of cognitive dysfunctions is recognized as a major public health problem. In fact, it has been estimated that the prevalence of dementia worldwide will increase from 115 in 2009 to 135 million patients in 2050 [1], becoming a serious burden for caregivers and generating high expenditures for the health care system [2]. The most common causes of cognitive impairment are Alzheimer’s disease (AD), vascular dementia, dementia with Lewy bodies, and frontotemporal dementia. Mild cognitive impairment (MCI), in contrast, is a syndrome characterized by a subtle decline in cognitive function and is considered a transitory state between normal aging and clinical dementia or AD. Progression rates for dementia and AD for adults with MCI vary from 6 to 25% per year, in comparison with 1 to 2% for the population without MCI [3]. The etiology of MCI and dementia is very complex and is based upon the interplay of genetic and environmental factors. Cognitive impairment is associated with impaired neuropsychiatric, physical, and social functioning, reducing quality of life, and predicting the development of dementia. Therefore, preventive strategies that protect individuals from neurological decline and minimize the development of related adverse effects are urgently needed [4,5]. Emerging evidence shows that early improvement of MCI decreases the prevalence of AD [6] and, in this regard, nutritional strategies are of special relevance [7,8]. Pathogenesis and progression of AD, the first cause of dementia, as previously stated, are linked to different inflammatory processes [9]. Of particular interest are the peripheral proinflammatory cytokines (interleukin (IL)-IL-1β, IL-6 and tumor necrosis factor (TNF)-α) observed in the peripheral blood and autopsy samples of patients with mild to moderate late onset of AD [10,11,12]. The pathologic hallmarks of AD are the presence of amyloid plaques, which consist primarily of a small peptide termed amyloid-β, and neurofibrillary tau tangles which have been used as a therapeutic target in drug development, although with limited success in clinical trials [13]. On the other hand, modest rates of brain atrophy are common in the elderly, due to normal aging; conversely, intermediate atrophy is observed in patients with MCI and also those with AD, who suffer from accelerated brain atrophy [14,15]. High rates of brain atrophy are characteristic of subjects with MCI that converts to AD [16]. The identification of factors that reduce the rate of atrophy seems to be an interesting approach to slow down this progression, since to date, no specific cure is available for AD [14,15]. In fact, the American Food and Drug Administration (FDA) has recently given its approval for Adulhem™ (adacanumab-avwa), the first new treatment approved for Alzheimer’s since 2003 and being the first therapy that targets the fundamental pathophysiology of the disease. Specifically, adacanumab-avwa action is focused on the reduction of amyloid-β plaques in the brain [17]. Folic acid (FA) is an essential nutrient, traditionally used for the management of macrocytic or megaloblastic anemia. Conventionally, FA is known owing to its key role in the prevention of Neural Tube Defects [18]. Additionally, it has been used to prevent the onset of megaloblastic anemia or to reverse it in the case of patients already diagnosed [19]. FA is also associated with the prevention of cancer (colorectal, lung, pancreatic, esophageal, cervical, breast, neuroblastoma, and leukemia) [20,21,22,23] and with improved immune system functioning [24]. Likewise, folate deficiency has been linked to neurocognitive dysfunctions [25]; the etiology of this risk factor is unclear, although it appears to be related to the associated hyperhomocysteinemia (HHcy) [26]. It should be highlighted that homocysteine (Hcy) is an intermediate in the methionine metabolism. In one-carbon metabolism, FA is a cofactor and vitamin B_12_ is a co-enzyme that promotes Hcy demethylation. Moreover, vitamin B_6_ is involved in Hcy conversion to cystathionine. Therefore, FA deficiency leads to increases in Hcy levels, the so-called HHCy. Moreover, vitamin B_6_ and B_12_ deficiencies can also cause HHCy [27], and this condition has been identified as an independent risk factor for cardiovascular and cerebrovascular disease [28]. Moreover, HHcy has also been reported as a risk factor for cognitive impairment, AD, and vascular dementia [25,29,30,31]. Low FA levels and HHCy have also been reported to be associated with brain and grey matter atrophy [32,33]. It has been estimated that approximately 69.8% of elders have HHcy (76.2% of the men and 66.4% of the women) [34]. Similar results were obtained in institutionalized older adults where the found HHcy prevalence was 63.0% [35]. Several potential mechanisms have been proposed to explain the deleterious effects of Hcy on cognitive function: DNA breakdown induced by Hcy, oxidative damage, an apoptotic process, and the excitotoxic cell dead owing to the direct activation of the neuronal N-methyl-D-aspartate (NDMA) receptor after homocysteic acid formation [36,37]. The influence of FA on cognition has been postulated since the 1970s [38]. However, to date, there has been some controversy about the efficacy of vitamin supplementation in patients with AD or MCI, as many of the available studies have yielded contradictory results. Therefore, the aim of this review is to summarize the current knowledge on the effects of dietary interventions based on the supplementation with FA, either alone or in combination with different vitamins or nutrients into the progression of AD and MCI. For a better understanding and follow-up, we have structured this review based on the nutrient combination used in each intervention, highlighting the cognition and biochemical markers employed for the evaluation of the disease progression.

## 2. Materials and Methods

### 2.1. Search Methods

Intervention studies from database initiation, up to and including 15 May 2020, were searched using the following bibliographic online databases: MEDLINE, PubMed, Scopus, and Google Scholar using the subsequent search terms: “supplementation”; “folic acid”; “folate”; “Alzheimer’s disease”, “cognitive impairment” AND “elderly”. Articles were restricted to those conducted in humans and published in English.

### 2.2. Selection Criteria and Eligibility

Eligible populations included both men and women, aged older than 50 years. Articles were eligible if they included FA supplementation either alone or in combination with other vitamins or nutrients. For the evaluation of the effectiveness of the intervention studies, cognitive function tests and blood biomarkers were selected as the main outcomes.

## 3. Interventions Based on Folic Acid Supplementation Alone

The first research studies to investigate the effect of FA supplementation on cognitive function was published in the early years of the 21st century (Table 1). The main limitation of the first published intervention studies was the limited number of patients analyzed. Specifically, Yukawa et al. [39] supplemented with FA (15 mg/day, 60 days) to 36 Japanese patients with low serum FA levels, selected among an entire sample of 343 neurological patients (mean age 57.0 ± 19.6 years). Vitamin administration improved neurological symptoms (assessed by electrophysiological studies, mental tests and neuroradiological examinations) in 24 of 36 cases (67%), especially in patients with neuropathy, while folate therapy was relatively more effective to biochemical biomarkers in neurological patients without dementia [40]. Later, Sommer et al. [40] published the results of a preliminary study conducted on 11 patients (>65 y) from the United States of America (USA) with dementia and low-normal FA levels which were treated with 10 mg/day of FA for 10 weeks. Supplementation effects were compared with placebo, and a battery of tests were administered for the evaluation of the cognitive function, including the Mini-Mental State Examination (MMSE), the Hamilton Rating Scale for Depression (HRSD), the Brief Psychiatric Rating Scale (BPRS), the Control Oral Word Formation, the Benton Visual Retention Test, the Boston Naming Test or the Trail Making test, among others. Preliminary results suggested that FA supplementation was not helpful for the improvement of symptomatology of patients with dementia [40]. The main limitation of these preliminary published reports was the limited number of participants in each study, which prevented the establishment of significant differences between intervention and control groups in terms of cognitive function scores. 

Since then, research available to date on the impact of FA supplementation alone on cognitive function and AD has been somewhat limited. One of the few studies available was undertaken in Chinese older adults aged 65 and older with MCI (*n* = 159) and unexposed to FA fortification and without previous supplementation. The study, of six months’ length, compared the effect of FA supplementation (400 µg/day) against conventional treatment without this vitamin. The test of cognitive performance (Full-Scale IQ score and the Chinese version of the Wechsler Adult Intelligence Scale—Revised (WAIS-RC), and the MMSE) as well as biochemical biomarkers (serum folate and vitamin B_12_, Hcy, and S-adenosylmethionine (SAM)) were measured at baseline and after 3 and 6 months of intervention. WAIS-RC included different subtests: Information, Similarities, Vocabulary, Comprehension, Arithmetic, Digit Span, Block Design, Picture Completion, Digit Symbol-Coding, Object Assembly, and Picture Arrangement for the neuropsychological assessment. Significant improvements in all biomarkers were established in the intervention group over the control group. Furthermore, FA supplementation improved the Full-Scale Intelligence quotient (IQ), the Digit Span and the Block Design scores vs. control at 6 months, confirming the potential of this nutritional intervention for the improvement of intellectual function [41]. Following the same research line, these authors then examined the association of a 12-month FA supplementation with changes in the cognitive performance, deepening in the role of peripheral inflammatory cytokines in this association. Thus, 168 elders from China (older than 65 y) with MCI were selected and randomly assigned to the intervention (FA 400 µg/day) and control subgroups. Peripheral inflammatory cytokines and biomarkers of folate status were determined at baseline and after 6 and 12 months of intervention. Cognitive function, the main outcome of the study, was measured by means of the WAIS-RC as well as with the calculation of the Full-Scale IQ at the same time points. FA supplementation resulted in an enhancement in biomarkers of folate status (serum FA and Hcy levels) and peripheral inflammatory cytokines (IL-6, TNF-α and Aβ-42 levels). Regarding cognitive status, improvements in the Full-Scale IQ and in the Information and Digit Span scores of WAIS-RC were found in the intervention group compared to control [42]. To note is that the information test is a valid indicator of long-term memory, whereas the Digit Span test examines attention and short-term memory [46,47]. Additionally, a significant reduction in the levels of peripheral inflammatory cytokines, including IL-6, TNF-α, circulating β-amyloid (Aβ)-42, as well as plasma Hcy concentration in the intervention group during the follow-up period was observed [42]. Therefore, results of this study confirmed the benefits of FA supplementation in global cognitive function of the elders, as suggested by the previous report. 

In order to continue exploring the effect of FA not only in the cognitive function, but also in the pathological mechanisms of MCI, Ma et al. [43] extended the intervention study with FA 400 µg/day up to two years. Hence, 180 Chinese individuals with MCI were included in the study and randomly distributed into intervention and control groups. Cognitive function was analyzed through the WAIS-RC and the full-scale IQ scores at the baseline and after 6, 12, 18, and 24 months. Moreover, blood Aβ-related biomarkers were also determined [43]. The interest of analyzing these biomarkers is based on the increasing evidence that suggests that epigenetic modifications, including DNA methylations, are involved in AD pathogenesis [48]. DNA methylation usually results in suppression of gene expression if this happens in its regulatory region. Folate is important for SAM production, which is converted to S-adenosylhomocysteine (SAH), as well as for thymidine and purines synthesis, the universal donor groups for DNA methylation. Alterations in the SAM/Hcy cycle (producing Hcy accumulation) are responsible for decreased SAM levels and, in turn, for reduced DNA methylation [49]. Likewise, currently, it is well-known that DNA methylation is involved in amyloid precursor protein (APP)-processing and in Aβ production, and that SAM is able to silence the gene involved in Aβ formation. Since reducing Aβ seems to be crucial for AD therapy, as an essential component of one-carbon metabolism, FA seems to be involved in the genetic regulation of the central nervous system by down-regulating DNA methylation [50]. Therefore, results of the intervention study [43] revealed significantly greater scores for the full-scale IQ, the verbal IQ, and the subtests of the Information and Digit Span of the WAIS-RC in the intervention subgroup compared to control. Regarding Aβ biomarkers, supplementation decreased plasma Hcy, SAH, and Aβ-42 levels and APP-RNA expression while increasing plasma SAM, the SAM/SAH ratio and DNA methyltransferase (DNMT) 1-mRNA and DNMT3a-mRNA expression, relative to the control group. These findings support the assumption that the neuroprotective role of folate may be associated to amyloidogenesis modulated by altered DNA methylation [43]. 

As mentioned elsewhere, low serum folate levels have been linked to cognitive decline. With the aim of elucidating the effects of FA short-term supplementation on patients with folate deficiency in cognitive impairment, an intervention study among Japanese elders attending to the dementia outpatient clinic of two different hospitals was carried out. Specifically, patients (*n* = 45; mean age 79.7 ± 7.9 y) with serum folate concentrations lower than 3.6 ng/mL were included in the study and supplemented with FA (5 mg/day). For the evaluation of the cognitive function, MMSE was carried out by clinical psychologists, and vitamins and Hcy levels were determined at the baseline and after 28 and 63 days of supplementation. Further MMSE follow-up was performed 6, 12, and 24 months later. Magnetic resonance imaging was employed for the analysis of the degree of hippocampal atrophy. After folate supplementation, Hcy levels were markedly reduced from 25.0 ± 18.0 to 11.0 ± 4.3 nmol/mL, whereas serum folate levels significantly increased from 2.7 ± 0.6 to 173.3 ± 257.2 ng/mL. Regarding the MMSE scores, a statistically significant change from 20.1 ± 4.7 to 22.2 ± 4.3 (*p* < 0.001) was observed. Of interest, it should be underlined that MMSE score improvement was positively correlated with Hcy level at the baseline and its reduction by FA supplementation; however, no significant correlations were found between the degree of MMSE change by folate supplementation and the degree of hippocampal atrophy [44]. Despite the fact that the mechanisms by which FA supplementation improve cognitive function remain unclear, it has been hypothesized that FA supplementation and lower Hcy levels might improve methionine synthase activity and D4 dopamine receptor-mediated phospholipid methylation, which have been reported to play a key role in attention and cognition [51]. 

Currently, the main drugs approved for AD treatment are cholinesterase inhibitors (donepezil, rivastigmine, and galantamine) and an N-methyl-D-aspartic acid receptor antagonist (memantine). Whilst they are useful for symptomatology management, they have little effect on the progression of the disease [52]. On the other hand, the FDA has recently approved adacanumab-avwa, a monoclonal antibody, as the first and only AD treatment that addresses the defining pathology of the disease by reducing amyloid-β plaques in the brain [17]. In this regard, the combination of therapeutic and nutritional strategies could be of interest. Connelly et al. [45] studied the effectiveness of the combination of a cholinesterase inhibitor and FA in AD patients from Scotland. To do so, patients with probable AD (according to the NINCDS-ADRDA criteria [53]) and a Modified Hachinski Ischaemic Scale score between 0 and 1 (due to the association between HHCy and cerebrovascular disease [54]) were selected and treated with a cholinesterase inhibitor and either FA (1 mg/day) or placebo. Specifically, 35 patients (mean age 76.27 ± 6.23 y) received donepezil, 12 rivastigmine, and 10 galantamine. No differences were observed in the different subgroups at the baseline in the MMSE score of Hcy levels. After 6 months of intervention, significant differences were detected in Hcy levels in those receiving FA vs. placebo. Moreover, significant changes from baseline were observed in the Activities of Daily Living (ADL) and Social Behavior scores between therapeutic arms (+1.5 (SD 5.32) vs. −2.29 (SD 6.16) in folate and placebo subgroups, respectively). However, no changes were found in MMSE scores at the end of the intervention [45]. These findings suggest that pharmacological and nutritional treatment may have a synergistic effect on the management of AD progression. Nevertheless, larger-scale studies are needed to confirm these findings and to obtain more consistent results. 

## 4. Interventions Based on the Combination of Folic Acid and Vitamin B_12_

Detailed intervention research studies based on the combination of FA and vitamin B_12_ have been carried out (Table 2). The first study that delved into the potential benefits of FA and vitamin B_12_ was performed on elder patients from Sweden with dementia and HHcy. The study included 33 subjects (mean age 78.4 ± 8.1 years), which were supplemented with a combination of 1 mg of vitamin B_12_ and 5 mg of FA daily for two months. Improvements in the MMSE and in the “Short cognitive performance test for assessing memory and attention” (SKT) scores were determined in patients with moderate dementia and elevated plasma Hcy levels, but not in those with severe dementia or normal plasma Hcy levels [55]. 

Late-life depression is associated with an increased risk of cognitive impairment [59]. Therefore, nutritional supplementation could be an interesting strategy for the management of depressive older adults. To delve deeper into this hypothesis, a randomized controlled trial was conducted in 900 Australian adults aged 60–74 years with elevated psychological distress, supplemented with FA and vitamin B_12_ (400 µg and 100 µg per day, respectively, formulated in a single tablet) for 2 years. The main outcomes for the examination of the cognitive function were the Telephone Interview for Cognitive Status-Modified (TICS-M), the Brief Test of Adult Cognition by Telephone (BTACT) for the measurement of processing speed, and the Informant Questionnaire on Cognitive Decline in the Elderly (IQCODE). Significant improvements after 2 years with FA and vitamin B_12_ supplementation were observed in TICS-M total, TICS-M immediate, and TICS-M delayed recalls scores in comparison with placebo. No significant changes were evidenced for TICS-M (including orientation, attention/calculation, or semantic memory), BTACT, or IQCODE. Moreover, lower Hcy levels were found at the end of the treatment in the supplemented group than in the control [56]. 

Eussen et al. [57] published the results of an intervention study carried out in The Netherlands among older adults (*n* = 195; ≥70 y) with mild vitamin B_12_ deficiency. Volunteers of this double-blind, placebo-controlled trial were randomly distributed in different groups and received high doses of this vitamin (1000 µg/day), either alone or in combination with FA (400 µg/day) or placebo for 24 weeks. Biochemical measurements (methylmalonic acid, Hcy and holotranscobalamin) were determined at baseline, and after 12 and 24 weeks of treatment, cognitive function (by means of the MMSE, the Clinical Dementia Rating (CDR) scale and the Geriatric Depression Scale (GDS)) was assessed at baseline and at the end of the supplementation. Oral supplementation with these vitamins did not improve cognitive function; in fact, only an improvement in memory function was observed in the placebo subgroup. Neither B_12_ alone, nor in combination with FA led to any improvements in any cognitive domains. According to the author’s opinion, this lack of effect of the supplementation could be associated to the hypothesis that patients with MCI for less than 6 months are more likely to respond to vitamin therapy than those with cognitive problems for more than 6 months, which may present widespread neurologic damage and loss of ability to repair neurons [60,61]. In this regard, the main limitation of this study is the lack of knowledge on the duration of the cognitive impairment in the patients [57]. 

As aforementioned, inflammatory processes are thought to play a crucial role in the pathogenesis of AD and other neuropsychiatric symptoms [62]. In this regard, Hcy may also induce inflammation by means of increasing oxidative stress or through nuclear factor kappa-β activation [63]. To elucidate the effect of FA and vitamin B_12_ on cognitive performance, through the reduction of peripheral inflammatory cytokine’s levels, a study was conducted with elders with MCI. Chinese volunteers aged 65 and over with MCI (*n* = 240) were randomly assigned to four treatment groups: supplemented with FA alone (800 µg/day), vitamin B_12_ alone (25 µg/day), FA plus vitamin B_12_ (800 µg/day plus 25 µg/day), or placebo. Both cognition function (measured using the WAIS-RC) and blood biomarkers were determined at baseline and after 6 months of supplementation. The obtained results revealed that the combination of FA and vitamin B_12_ in elders significantly improved cognitive performance and reduced levels of proinflammatory cytokines (including IL-6, TNF-α and MCP-1) in human peripheral blood, compared with controls. Specifically, supplementation with both vitamins changed Full-Scale IQ, verbal IQ, and Information and Digit Span scores of the WAIS-RC. Interestingly, results attained with the vitamins combination were significantly greater than either FA or vitamin B_12_ alone [58]. 

## 5. Interventions Based on the Combination of Folic Acid and Vitamins B_6_ and B_12_

As previously stated, HHcy has been associated with cognitive impairment and dementia [25,30]. In order to elucidate the effect of vitamin supplementation on cognitive function and on lowering Hcy levels, Cheng et al. [64] carried out a study in middle-aged and elderly Chinese individuals (aged 55 to 94 years) with HHcy. Fifty-seven patients were included in the intervention group and supplemented with daily oral doses of a combination of 800 µg of FA, 10 mg of vitamin B_6_, and 25 µg of vitamin B_12_ for 14 weeks, whereas the remaining 47 patients were included in the control group and received a placebo capsule daily during the same timeframe. Patients’ cognitive function was evaluated by means of the Basic Cognitive Aptitude Tests (BCATs). The BCATs scores of the intervention group significantly increased compared to control, indicating an improvement of the cognitive function, especially for the consciousness speed, spatial image ability, digit working memory ability, and figure memory ability. Moreover, as expected, supplementation resulted in a significant reduction of Hcy levels [64]. 

To date, the potential of B-vitamins to reduce high plasma total Hcy levels (i.e., HHcy) have led to controversial results (Table 3). In order to clarify this eventual effect, an intervention study was conducted in 8164 patients from 20 countries on five continents with previous stroke or transient ischemic attack. Six months after the qualifying stroke, participants fulfilled the MMSE, and 38% (*n* = 3089 participants) were selected as cognitively unimpaired. These patients were then randomly allocated to double-blind treatment with one tablet daily containing 2 mg of FA, 25 mg of vitamin B_6_ and 500 µg of vitamin B_12_ or placebo, and followed up for 2.8 years. At the end of the study, it was observed that the supplemented subgroup, compared with placebo, showed a reduction in Hcy plasma levels, but no effect was observed in either of the MMSE scores. Likewise, no differences were observed in terms of the incidence of cognitive impairment or the rate of cognitive decline [65]. Nevertheless, it is worth highlighting that MMSE is susceptible to ceiling effects in high-functioning populations and has a low sensitivity for cognitive impairment [66,67]. 

HHcy and cognitive impairment commonly affect kidney transplant recipients [76]. Despite Hcy levels frequently falling after kidney transplantation, HHcy typically persist, remaining in levels associated with increased risk of cerebrovascular disease and cognitive decline [77]. In this sense, Scott et al. [68] carried out a study in kidney transplant recipients from the USA, Canada, and Brazil (*n* = 584; aged 35 to 75 years, mean age 57.2 years), with stable kidney function for at least six months after transplantation and high Hcy levels. Participants of the treatment group received a daily multivitamin containing high doses of folate, vitamin B_12_ and vitamin B_6_ (5 mg, 1 mg and 50 mg, respectively) whereas the placebo group took a daily multivitamin without folate and vitamin B_12_ and B_6_, consistent with the recommended daily allowances (2 µg and 1.4 mg, respectively). Mandatory FA fortification of flour affected all participants. Cognitive testing included tests that evaluate different domains of cognition and mood, such as verbal memory (Word List Learning), executive function, and processing speed (Trails A&B and Digit Symbol Coding), construction and reasoning (Block Design), and depression (Center for Epidemiological Studies-Depression, CES-D). Participants were tested at baseline and, on average, after 3.3 years. Biomarker analysis included plasma Hcy levels, as well as vitamin levels. At baseline, cognitive impairment prevalence was 61%. Supplementation provided a significant increase in processing speed and memory scores in the supplemented group compared to control. No interactions were detected between the Hcy level at baseline, B vitamin status, and treatment on the cognitive outcomes. Noteworthily, the majority of the participants were folate- and vitamin B_12_-sufficient at baseline. Therefore, the authors concluded it could be of interest to analyze the potential benefits of B-vitamin therapy in individuals with inadequate B-vitamin status [68]. 

Ford et al. [69] carried out an intervention study with 299 hypertensive Australian men aged 75 years and older for two years in order to compare supplementation with FA, vitamin B_6_ and vitamin B_12_ (2 mg, 25 mg and 400 µg per day, respectively) with placebo in terms of cognitive function. To do so, researchers selected as the primary outcome of interest the Alzheimer’s Disease Assessment Scale (ADAS), specifically, the cognitive subscale (ADAS-cog). Moreover, researchers evaluated the risk of cognitive impairment and dementia over eight years. No significant changes were detected in ADAS-cog from baseline to the end of the study between the supplemented and control group, indicating that the use of these vitamins did not change the rate of cognitive decline. Certain changes not sustainable over time were observed on measures of immediate recall and attention. In addition, supplementation did not seem to reduce the risk of cognitive impairment or later diagnosis of dementia [69]. In relation to this study, it should be noted that authors selected men since they have higher Hcy levels than women as do hypertensive people compared with normotensive ones. At the end of the study, Hcy levels decreased by 22.5% in the intervention group compared with a 10.7% increase in the placebo group, whereas amyloid-β peptide levels were lower in the intervention group than in controls (7.0 pg/mL vs. 26.8 pg/mL), indicating the potential role of B-vitamins in AD prevention [78]. 

Brain atrophy, in different degrees, is characteristic of patients with MCI or AD [14], and Hcy seems to be a risk factor for brain atrophy. Therefore, in order to determine if FA and vitamins B_6_ and B_12_ supplementation could slow down the rate of brain atrophy by means of Hcy reduction, a randomized controlled trial was carried out with 271 volunteers from the United Kingdom (UK) who were older than 70 years with MCI. Participants were assigned to an intervention (0.8, 0.5 and 20 mg/day of FA and vitamins B_12_ and B_6_, respectively) or control group (placebo); the study lasted 2 years. Supplementation with Hcy-lowering vitamins resulted in a significant brain atrophy deceleration (mean rate atrophy per year 0.76% vs. 1.08% in control). In addition, this greater atrophy rate was associated with lower final cognitive test scores. Moreover, there was an interaction between supplementation and Hcy levels at baseline [70]. According to obtained results, it would be of interest to analyze the effects of this treatment in the cognitive function of patients with AD to study the potential delay in disease progression. It should be highlighted that this trial was mainly focused on the detection of changes in atrophy and not into the cognition-related benefits. In the second stage of their research, the same authors analyzed, on the same subjects, the effect of B-vitamin supplementation on gray matter cerebral atrophy, specifically in those regions vulnerable to the AD process. Results of this study demonstrated that supplementation reduced seven-fold the atrophy in specific brain regions, including the temporal lobe. Furthermore, in the control group, higher Hcy levels at the baseline were linked to faster gray matter atrophy. However, this effect was prevented by B-vitamin supplementation. In addition, researchers confirmed the association between the reduction of Hcy levels by these B vitamins, which resulted in a decrease in gray matter atrophy, which, in the end, led to the slow-down of cognitive decline [71]. 

Regular physical exercise is linked to improvements in cognitive function and a delay in the occurrence of AD [79]. Furthermore, it has been shown to enhance cognitive function in both cognitively healthy older adults and those with dementia [80]. A randomized placebo-controlled trial was designed to examine the effect of a double intervention: physical exercise (a moderate-intensity walking program) followed by supplementation (daily vitamin tablet with 5 mg of FA, 0.4 mg vitamin B_12_ and 50 mg of vitamin B_6_) on the cognitive function of Dutch older adults (*n* = 152, 70–80 y) with MCI. Results reported the lack of effectiveness of the double-intervention program on improving cognitive function of the volunteers. Cognitive tests were performed at baseline and at different time points during the intervention: the MMSE, the Auditory Verbal Learning Test (AVLT), the Verbal Fluency Test (VFT), the Digit Symbol Substitution Test (DGST), and the Abridged Stroop Color Word Test (SCWT-A) in order to assess different aspects of cognition, such as memory, information processing of attention. Compared to placebo, only significant differences were observed after the physical exercise program in memory and attention items [72]. These results could be attributed to the short intervention length (1 year each). Longer interventions and follow-up might provide a better insight into the potential beneficial effects of these programs on cognitive impairment.

Similarly, another study deepened into the potential of multivitamin supplementation on the improvement of AD cognitive symptoms, in patients treated with an acetylcholinesterase inhibitor. Hence, male and female patients from Taiwan (*n* = 89), with mild to moderate AD and aged more than 50 years, were included in this 26-week, randomized, double-blind, and placebo-controlled clinical trial. All enrolled patients were prescribed donepezil and randomly received either a multivitamin oral supplement (containing 0.5 mg of mecobalamin, 1 mg of FA and 5 mg of vitamin B_6_ as well as different amounts of ferrous iron, nicotinamide, calcium carbonate, iodine, copper, ascorbic acid, vitamin B_12_ and vitamin D_3_, among others) or placebo daily. Hcy levels were determined at baseline and at the end of the study. As the primary efficacy outcome, changes in the score of the ADAS-cog (11 items) were calculated. Secondary efficacy outcomes included ADL function, changes in MMSE, the Cognitive Abilities Screening Instrument and the Instrumental ADL Scale. Supplementation was associated with a significant mean descent in Hcy levels. Nevertheless, no significant differences were detected in cognition or ADL functions between subgroups, even after stratification by age or gender. Moreover, surprisingly, no associations between changes in cognition and Hcy levels were found. According to the author’s conclusions, these results could be attributed to the short duration of the intervention and the high number of patients who dropped out owing to the adverse effects, such as muscle pain or insomnia, which were encountered [73]. 

A longer study was conducted by the AD Cooperative Study, a consortium funded by the National Institute on Aging of the USA. Researchers recruited individuals (*n* = 340) with probable AD that met the following inclusion criteria: age greater than 50 and MMSE score between 14 and 26. Patients under chronic treatment (more than 3 months) with an acetylcholinesterase inhibitor or memantine were also included. Intervention consisted of the administration of a tablet containing FA (5 mg), vitamin B_6_ (25 mg), and vitamin B_12_ (1 mg) daily, or placebo for 18 months. Plasma Hcy and vitamin B_6_ levels were analyzed at different time points. Likewise, ADAS-cog was selected as the primary outcome, whereas secondary outcome measures comprised the MMSE, Clinical Dementia Rating sum of boxes (CDR-SOB), AD Cooperative Study ADL (ADCS-ADL) scale, Neuropsychiatric Inventory, Quality of Life-AD, and the time of attainment of significant endpoints (4-point decline from baseline ADAS-cog score, death, institutionalization, a one-stage worsening on the global CDR scale, 15-point decline on the ADCS-ADL). The intervention was successful in reducing Hcy levels, but no evidence of benefit of any outcome measure was detected when considering the population as a whole. Specifically, in the study subgroup, Hcy levels decreased by 31%, but no impact was observed in any of the measured outcomes. Thus, for example, the change in ADAS-cog in the supplemented subgroup was 0.401 points/month and 0.372 points/month in placebo [74]. 

The protective role of ω-3 fatty acids in cognitive impairment and dementia is also somewhat a matter of controversy. It has been postulated that plasma ω-3 fatty acid concentrations (specifically eicosapentaenoic acid (EPA) and docosahexaenoic acid (DHA)) could modify the effect of Hcy-lowering B vitamins supplementation on brain atrophy rates. To confirm this relationship, Jernerén et al. [75] conducted a study with 168 patients from the UK with MCI (≥70 y). Patients were supplemented with B-vitamins daily (FA, 0.8 mg; vitamin B_6_, 20 mg and vitamin B_12_, 0.5 mg) or placebo for two years, and cranial MRI scans were performed at baseline and at the end of the intervention. High plasma EPA and DHA levels seemed to play a crucial role in supplementation effectiveness. Thus, in patients with high levels of ω-3 fatty acids at baseline (>590 µmol/L), B-vitamins reduced the mean atrophy rate by 40.0% compared to placebo (*p* = 0.023), while no significant impact on the atrophy rate was shown among subjects with low baseline levels of these lipids (<390 µmol/L) [75]. These findings emphasize the relevance of identifying subgroups that may benefit from future clinical trials. 

Neuroinflammation and oxidative stress play a critical role in the pathogenesis of dementia [81]. Different parameters have been approached as biomarkers of inflammation and/or oxidative stress, including malondialdehyde, tryptophan, neopterin kynunerine or carbonyl proteins. Both tryptophan and kynunerine are involved in the serotonin pathway and in cognition procedures [82,83]. In fact, low tryptophan levels have been detected in demented patients, and the ratio of kynunerine/tryptophan is of great interest as a prognostic marker for dementia [84]. Malondialdehyde and carbonyl proteins are well-known markers of oxidative stress [85,86]. Carbonyl proteins are increased in both the cerebrospinal fluid and plasma of patients with neurodegenerative and neuro-inflammatory diseases [86,87]. Rommer et al. [88] assessed the influence of vitamin supplementation on parameters of oxidative stress, inflammation, and cognition in patients from Austria with AD and MCI. Supplementation was performed as follows: 1 tablet/day for 1 month, followed by 3 tablets/day for another month, and finally, 2 tablets/3 times a day for another month. Each tablet contained 50 mg of vitamin B_1_, 50 mg of vitamin B_6_, 5 mg of FA, and 0.05 mg of vitamin B_12_. Thus, 48 individuals enrolled in the study, and were divided into three subgroups: healthy controls without supplementation, AD patients without supplementation, and supplemented AD patients. MMSE and laboratory biomarkers (carbonyl proteins, malondialdehyde, tryptophan, kynunerine, neopterin, FA, and vitamin B_12_ levels) were selected as the main outcomes. The MMSE score was greater in the supplemented group, despite no significant differences being found in comparison to patients without supplementation. Moreover, levels of carbonyl proteins were significantly greater in non-supplemented patients, whereas both tryptophan levels and the kynunerine/tryptophan ratio were lower in this same subgroup compared to supplemented patients [88]. The main interest of these findings was the interest of carbonyl proteins as a potential suitable biomarker for monitoring demented patients (Table 4). 

Combinations of vitamins with other nutrients, such as ω-3 fatty acids, have been evaluated as an interesting strategy for the management of patients with MCI (Table 4). Specifically, recently published research explored the effects of 6-months supplementation with FA (0.8 mg/day) and docosahexaenoic acid (DHA) (800 mg/day) either alone or combined, in comparison with placebo in 160 Chinese patients aged more than 60 years with MCI. Blood Aβ biomarker levels, whose production is involved in the pathological cascades of AD, were assessed at the baseline and at the end of the intervention. Moreover, the patient’s cognitive function was also determined at these same time points and after 12 months. Both FA, DHA, and their combination improved cognitive function (measured by the full-scale intelligence quotient (FSIQ) and other arithmetic, picture complement and digit span scores) compared to placebo. Likewise, supplementation with either FA or the combination of FA with DHA led to a reduction in Hcy levels, whereas FA plus DHA treatment reduced levels of Aβ biomarkers (Aβ40 and Aβ42) [89]. According to the analyzed studies, further research is needed on the effects of nutrient combination to slow down the progression of cognitive impairment. 

## 6. Conclusions

Altogether, the available results suggest that nutritional interventions may play an important role in the progression of cognitive impairment and Alzheimer’s disease. However, there is a strong need to clarify the optimal supplementation length that leads to measurable benefits by means of the available cognitive function tests. Moreover, other factors, such as vitamin dosage or their combinations and the target population, seem to be important factors that could explain the observed discrepancies among the studies published to date. Moreover, Hcy levels could be a predictable factor that may influence the success of the intervention. Undoubtedly, it is crucial to keep investigating the benefits of nutritional interventions on these diseases, considering the great economic impact as well as the potential of developing effective therapies that seems crucial to improve the quality of life of these patients and the whole society.

## Figures and Tables

**Table 1:** Summary of the interventions based on folic acid.

Study Subjects	Intervention	Outcomes	Effects	Author and Year
Neurological patients from Japan (*n* = 36; mean age 57.0 ± 19.6 y) with low serum FA levels.	FA (15 mg/day) for 60 days.	-Cognitive function: electrophysiological studies, mental tests and neuroradiological examinations. -Biomarkers: serum FA levels.	-Improvement of neurological symptoms, especially in patients with neuropathy -Increase in folate levels in neurological patients without dementia	Yukawa et al., 2001 [39].
Patients from the USA (*n* = 11; >65 y) with dementia and low-normal FA levels	FA (10 mg/day) for 10 weeks vs. placebo.	-Cognitive function: MMSE, HRSD, BPRS, Control Oral Word Formation, Benton Visual Retention Test, Boston Naming Test and Trail Making test.	-FA supplementation does not improve symptomatology of patients with dementia.	Sommer et al., 2003 [40].
Chinese older adults with MCI (*n* = 159) aged 65 y and older.	FA (400 µg/day) vs. control for 6 months.	-Cognitive function: Full Scale IQ score, WAIS-RC and MMSE. -Biomarkers: serum folate and vitamin B_12_, Hcy, and S-adenosylmethionine.	-Improvement in the Full Scale IQ and in the Digit Span and Block Design scores of the WAIS-RC. -Significant improvements of all biomarkers in the intervention groups vs. control.	Ma et al., 2016 [41].
Chinese elders (*n* = 168) older than 65 y with MCI.	FA (400 µg/day) vs. control for 12 months.	-Cognitive function: WAIS-RC and Full Scale IQ score. -Biomarkers: serum FA and Hcy and peripheral inflammatory cytokines (IL-6, TNF-α and Aβ-42).	-Improvement in the Full Scale IQ and in the Information and Digit Span scores of WAIS-RC. -Enhancement of biomarker of folate status and peripheral inflammatory cytokines.	Ma et al., 2016 [42].
Chinese elders (*n* = 180) older than 65 y with MCI.	FA (400 µg/day) vs. control for 24 months.	-Cognitive function: WAIS-RC and Full Scale IQ score. -Biomarkers: blood Aβ related biomarkers	-Greater scores of full-scale IQ, verbal IQ and the Information and Digit Span subtests of the WAIS-RC. -Decrease in plasma Hcy, SAH and Aβ-42 levels and APP-RNA expression. -Increase in plasma SAM, SAM/SAH ratio and DNMT1-mRNA and DNMT3a-mRNA expression.	Ma et al., 2019 [43].
Japanese elders (*n* = 45; mean age 79.7 y) with dementia and low serum folate concentrations.	FA (5 mg/day).	-Cognitive function: MMSE -Biomarkers: folate and Hcy levels -Magnetic resonance imaging	-Increase of the MMSE score. -Increase in serum folate levels and decrease in Hcy levels.	Hama et al., 2020 [44].
Scottish patients with AD (*n* = 57; mean age 76.2 y) under treatment with cholinesterase inhibitors.	FA (1 mg/day) or placebo for 6 months.	-Cognitive function: MMSE and ADL and Social Behaviour score -Biomarkers: Hcy levels.	-Improvement in the ADL and Social Behaviour score in the intervention group vs. control. -No changes observed in MMSE. -Decrease of Hcy Levels in the intervention group vs. control.	Connelly et al., 2008 [45].

Aβ: Amyloid-β; ADL: Activities of Daily Living; APP: amyloid precursor protein; BPRS: the Brief Psychiatric Rating Scale; DNMT: DNA methyltransferase; FA: folic acid; Hcy: total homocysteine; HRSD: Hamilton Rating Scale for Depression; IL-6: Interleukin 6; IQ: Intelligence Quotient; MCI: mild cognitive impairment; MMSE: Mini-Mental State Examination; mRNA: messenger ribonucleic acid SAM: S-adenosylmethionine; SAH: S-adenosylhomocysteine; SKT: A short cognitive performance test for assessing memory and attention; TNF-α: tumor necrosis factor-α; WAIS-RC: Wechsler Adult Intelligence Scale-Revised in China.

**Table 2:** Summary of the interventions based on folic acid and vitamin B_12_.

Study Subjects	Intervention	Outcomes	Effects	Author and Year
Elder patients from Sweden with dementia and HHcy (*n* = 33; mean age 78.4 ± 8.1 y)	FA (5 mg/day) and vitamin B_12_ (1 mg/day) for two months	-Cognitive function: MMSE and SKT scores -Biomarkers: Hcy	-Improvements in cognitive function scores in patients with moderate dementia and elevated plasma Hcy levels. -No improvements in patients with severe dementia or normal plasma Hcy levels.	Nilsson et al., 2001 [55].
Australian adults (*n* = 900) aged 60–74 y with elevated psychological distress	FA (400 µg/day) and vitamin B_12_ (100 µg/day) for 2 y vs. control	-Cognitive function: TICS-M, BTACT and IQCODE -Biomarkers: Hcy	-Improvements in TICS-M total, TICS-M immediate and TICS-M delayed recalls scores compared with placebo -No changes detected for TICS-M, BTACT or IQCODE in the intervention group vs. control -Decrease in Hcy levels vs. placebo	Walker et al., 2012 [56].
Dutch old adults (*n* = 195; ≥70 y) with mild vitamin B12 deficiency.	Vitamin B_12_ (1000 µg/day) alone or in combination with FA (400 µg/day) or placebo for 24 weeks	-Cognitive function: MMSE, CDR scale and GDS	-No improvements detected after oral supplementation in any cognitive domain	Eussen et al., 2006 [57].
Chinese volunteers (*n* = 240) aged 65 y and over with MCI	FA alone (800 µg/day), vitamin B_12_ alone (25 µg/day), FA plus vitamin B_12_ (800 µg/day plus 25 µg/day) or placebo for 6 months	-Cognitive function: WAIS-RC -Biomarkers: IL-6, TNF-α	-Improvement of the cognitive performance Full Scale IQ, verbal IQ, Information and Digit Span scores of the WAIS-RC in patients treated with the combination of FA and vitamin B_12_. -Reduction of proinflammatory cytokines levels (including IL-6, TNF-α and MCP-1) compared with vitamins alone or control	Ma et al., 2019 [58].

BTACT: Brief Test of Adult Cognition by Telephone; CDR: Clinical Dementia Rating; FA: folic acid; GDS: Geriatric Depression Scale; Hcy: total homocysteine; IQCODE: Informant Questionnaire on Cognitive Decline in the Elderly; MCI: Mild Cognitive Impairment; SKT: Short cognitive performance test for assessing memory and attention; TICS-M: Telephone Interview for Cognitive Status-Modified; WAIS-RC: Wechsler Adult Intelligence Scale-Revised in China.

**Table 3:** Summary of the interventions based on folic acid and vitamins B_6_ and B_12_.

Study Subjects	Intervention	Outcomes	Effects	Author and Year
Chinese adults (*n* = 57) aged 55 to 94 y with HHCy.	FA (800 µg/day) of, vitamin B_6_ (10 mg/day) and of vitamin B_12_ (25 µg/day) for 14 weeks vs. control.	-Cognitive function: BCATs. -Biomarkers: Hcy.	-Increase of the BCATs scores of the intervention group vs. control. -Reduction in Hcy levels in the intervention group.	Cheng et al., 2016 [64].
Cognitively unimpaired patients (*n* = 8164) from 20 countries on five continents with previous stroke or transient ischemic attack.	FA (2 mg/day), vitamin B_6_ (25 mg/day) and vitamin B_12_ (500 µg/day) or placebo for 3.4 y.	-Cognitive function: MMSE -Biomarkers: Hcy	-Supplementation had no effect on MMSE scores. -Supplementation did not affect cognitive impairment or cognitive decline incidence. -Reduction in Hcy levels in the intervention group.	Hankey et al., 2013 [65].
Kidney transplant recipients from the USA, Canada and Brazil with high Hcy levels (*n* = 584; mean age 57.2 y).	High doses of FA (5 mg/day), vitamin B_6_ (50 mg/day) and vitamin B_12_ (1 mg/day) vs. no FA, vitamin B_12_ (2 µg/day) and B_6_ (1.4 mg/day).	-Cognitive function: Word List Learning, Trails A&B and Digit Symbol Coding, Block Design and CES-D tests. -Biomarkers: Hcy and vitamin levels.	-Significant increase in processing speed and memory scores in the supplemented group. -No interactions between Hcy level at the baseline, B vitamin status and treatment on the cognitive outcomes.	Scott et al., 2017 [68].
Hypertensive men from Australia (*n* = 299) aged 75 y and older.	FA (2 mg/day), vitamin B_6_ (25 mg/day) and vitamin B_12_ (400 µg/day) vs. placebo for 2 y.	-Cognitive function: ADAS-cog. -Biomarkers: Hcy.	-No differences in ADAS-cog between subgroups -Improvements, not sustainable over time, on immediate recall and attention measurements. -Decrease in Hcy levels in the intervention group vs. control.	Ford et al., 2010 [69].
Adults from the UK (*n* = 271) older than 70 y with MCI.	FA (0.8 mg/day), vitamin B_6_ (20 mg/day) and vitamin B_12_ (0.5 mg/day) vs. placebo for 2 y.	-Cognitive function: MMSE and TICS-M. -Biomarkers: folate and vitamin B_12_ biomarkers. -MRI scan.	-Brain atrophy deceleration. -Decrease in Hcy levels compared to the baseline. -Association between greater rate of atrophy and lower final cognitive test. -Interaction between treatment and Hcy levels at the baseline	Smith et al., 2010 [70].
Adults from the UK older than 70 y with MCI (*n* = 271).	FA (0.8 mg/day), vitamin B_6_ (20 mg/day) and vitamin B_12_ (0.5 mg/day) vs. placebo for 2 y.	-Biomarkers: Hcy. -MRI scan.	-Reduction on gray matter atrophy, including in the temporal lobe. -High Hcy level at the baseline associated with greater atrophy rates. -Prevention of gray matter atrophy by B-vitamin supplementation. -Decrease in gray matter atrophy, slow-down cognitive decline.	Douaud et al., 2013 [71].
Dutch older adults (*n* = 152; 70–80 y) with MCI.	Moderate intensity walking program (1 y) followed by FA (5 mg/day), vitamin B_6_ (50 mg/day) and vitamin B_12_ (0.4 mg/day) for 1 year vs. control.	-Cognitive function: MMSE, AVLT, VFT, DGST and SCWT-A.	-Improvement in memory and attention items after the physical exercise program.	van Uffelen et al., 2008 [72].
Taiwanese adults older than 50 y with mild to moderate AD *n* = 89).	Donepezil and randomly received either a multivitamin oral supplement (FA (1 mg/day), vitamin B_6_ (5 mg/day), mecobalamin (0.5 mg/day) and different amounts of other vitamins and minerals) or placebo for 26 weeks.	-Cognitive function: ADAS-cog, ADL function, MMSE, Cognitive Abilities Screening Instrument and Instrumental ADL Scale. -Biomarkers: Hcy.	-No differences detected in cognition or ADL functions between subgroups. -Descent in Hcy levels. -No associations found between changes in cognition and Hcy levels.	Sun et al., 2007 [73].
Patients from the USA (*n* = 340) with probable AD older than 50 y.	FA (5 mg/day), vitamin B_6_ (25 mg/day) and vitamin B_12_ (1 mg/day) vs. placebo for 18 months.	-Cognitive function: ADAS-cog, CDR-SOB, ADCS-ADL scale Neuropsychiatric Inventory, Quality of Life-AD and the time of attainment of significant endpoints. -Biomarkers: Hcy.	-No significant improvements detected in cognitive function. -Decrease in Hcy levels in the intervention group compared to control.	Aisen et al., 2008 [74].
Older adults from the UK (*n* = 168) with MCI aged more than 70 y.	FA (0.8 mg/day), vitamin B_6_ (20 mg/day) and vitamin B_12_ (0.5 mg/day) vs. placebo for 2 y.	-Biomarkers: EPA and DHA. -MRI scan.	-Reduction in mean atrophy rate in patients with high EPA and DHA levels at the baseline.	Jernerén et al., 2015 [75].

AD: Alzheimer’s Disease; ADAS-cog: Cognitive subscale of the Alzheimer’s Disease Assessment Scale; ADCS-ADL: Alzheimer’s Disease Cooperative Study of Activities of Daily Living; ADL: Activities of Daily Living; AVLT: Auditory Verbal Learning Test; BCATs: Basic Cognitive Aptitude Tests; CES-D: Center for Epidemiological Studies-Depression; CDR-SOB: Clinical Dementia Rating sum of boxes; DHA: docosahexaenoic acid; DGST: Digit Symbol Substitution Test; EPA: Eicosapentaenoic Acid; Hcy: homocysteine; HHCy: hyperhomocysteinemia; MMSE: Mini-Mental State Examination; MRI: Magnetic Resonance Scanning; SCWT-A: Abridged Stroop Color Word Test; TICS-M: Telephone Interview for Cognitive Status-Modified; VFT: Verbal Fluency Test; 6. Interventions based on folic acid combined with other different nutrient combinations.

**Table 4:** Summary of the interventions based on folic acid combined with other nutrients.

Study Subjects	Intervention	Outcomes	Effects	Author and Year
Patients from Austria (*n* = 48) with MCI and AD.	FA (5 mg/day), vitamin B_6_ (50 mg/day), vitamin B_12_.(5 mg/day) and vitamin B_1_ (50 mg/day) 1 tabled/day for 1 month, followed by 3 tablets/day for another month and 2 tablets/3 times a day by another month	-Cognitive function: MMSE. -Biomarkers: carbonyl proteins, malondialdehyde, tryptophan, kynunerine, neopterin, FA and vitamin B_12_ levels.	-Greater MMSE score in the intervention subgroup vs. control. -Carbonylprotein levels were significantly greater in non-supplemented patients. -Tryptophan levels and the kynunerine/tryptophan ratio were lower in non-supplemented patients.	Rommer et al., 2016 [88].
Chinese adults (*n* = 160) older than 60 y with MCI.	FA (0.8 mg/day) and DHA (800 mg/day) alone or in combination for 12 months.	-Cognitive function: FSIQ and other arithmetic, picture complement and digit span scores. -Biomarkers: Aβ related biomarkers and Hcy.	-Both FA, DHA and FA + DHA combinations improved cognitive function vs. placebo. -FA and FA + DHA reduced Hcy levels. -FA + DHA reduced Aβ40 and Aβ42 levels.	Bai et al., 2021 [89].

Aβ: Amyloid β; AD: Alzheimer’s Disease; DHA: docosahexaenoic acid; FA: Folic Acid; FSIQ: full-scale intelligence quotient; Hcy: homocysteine; MCI: Mild Cognitive Impairment; MMSE: Mini-Mental State Examination.
